# Pregnancy Status Is Associated with Lower Hemoglobin A1c among Nondiabetes Women in the United States from NHANES 2005–2016

**DOI:** 10.1155/2022/4742266

**Published:** 2022-01-24

**Authors:** Yi Lu, Zhenyu Huo, Fan Ge, Jiachun Luo

**Affiliations:** ^1^Nanshan School, Guangzhou Medical University, Guangzhou, China; ^2^First Clinical College, Guangzhou Medical University, Guangzhou, China; ^3^Key Laboratory of Molecular Target & Clinical Pharmacology and the State Key Laboratory of Respiratory Disease, School of Pharmaceutical Sciences & the Fifth Affiliated Hospital, Guangzhou Medical University, Guangzhou, China

## Abstract

**Background:**

It has been verified that the incidence rate of diabetes mellitus (DM) is sharply increased in pregnant female adults. However, the relationship between pregnant status and hemoglobin A1c (HbA1c) in nondiabetes women remains unclear.

**Methods:**

We conducted a cross-sectional study of 7762 participants in the National Health and Nutrition Examination Survey (NHANES) 2005–2016. Multivariable linear regression models were performed to evaluate the associations between pregnant status with HbA1c and serum glucose in nondiabetes women.

**Results:**

HbA1c was significantly lower in the pregnant group than in the nonpregnant group. There was a negative association between urine pregnancy test and HbA1c in all three models (model 1: *β* = −0.23, 95% CI: (−0.18 to −0.27); model 2: *β* = −0.20, 95% CI: (−0.15 to −0.24); model 3: *β* = −0.24, 95% CI: (−0.20 to −0.29)). In the subgroup analysis stratified by age, this negative association existed in all age subgroups (age <20: *β* = −0.20, 95% CI: (−0.04 to −0.27); age ≥20, <35: *β* = −0.24, 95% CI: (−0.20 to −0.29); age ≥35: *β* = −0.28, 95% CI: (−0.17, −0.39)). In the subgroup analysis stratified by race, the negative associations steadily existed in different subgroups (Mexican American:*β* = −0.20, 95% CI:(-0.11 to -0.29); Other Hispanic:*β* = -0.31, 95% CI: (-0.16 to -0.46); Non-Hispanic White: *β* = −0.24, 95% CI: (−0.17 to −0.31); Non-Hispanic Black: *β* = −0.21, 95% CI: (−0.12 to −0.31); Other races:*β* = −0.22, 95% CI: (−0.08 to −0.35)). On the other hand, a negative association between self-reported pregnant status and HbA1c was also found (model 1: *β* = −0.22, 95% CI: (−0.18 to −0.27); model 2: *β* = −0.19, 95% CI: (−0.15 to −0.2); model 3: *β* = −0.23, 95% CI: (−0.19 to −0.28)). In the subgroup analysis stratified by age, this negative association also existed in all age subgroups.

**Conclusions:**

The study indicated that nondiabetes women with pregnant status had significantly lower HbA1c compared with those nonpregnant. Moreover, the negative associations between pregnant status and HbA1c steadily existed in subgroups stratified by age and gender.

## 1. Introduction

Currently, increasing data focusing on the correlation between hyperglycemia and pregnancy has been continuously issued. The global prevalence of hyperglycemia in pregnant women over 20 years of age is 15.8%, and more than 20 million pregnant women suffer from this disease every year [1]. Hyperglycemia during pregnancy is usually divided into three types: gestational diabetes mellitus (GDM), overt gestational diabetes mellitus (ODM), and prepregnancy diabetes mellitus (PDM) [2]. Both diabetes and prediabetes in pregnant women have been shown to be associated with many serious complications, such as miscarriage, stillbirth, and increased perinatal mortality [3, 4]. Therefore, it is very important to identify pregnant women with prediabetes and take early interventions to delay or even prevent the occurrence of diabetes.

HbA1c is widely used for the diagnosis of diabetes mellitus or glycemic control [5]. It was selected as a diagnostic and monitoring tool for diabetes in 2011 by the WHO [6]. However, HbA1c has not been used for GDM diagnosis until now. According to WHO criteria, HbA1c is not recommended for the diagnosis of GDM at present, while oral glucose tolerance test (OGTT) results are recommended [7]. Previous studies from Japan have demonstrated that HbA1c tends to be lower during pregnancy [8] but increases in late pregnancy [9]. Whether HbA1c tends to be lower or higher at different stages during pregnancy is a controversial topic. Further studies are required to clarify this issue. Studies on the clinical usefulness of HbA1c for GDM diagnosis and prediction of PDM development are ongoing. Moreover, there is increasing evidence on the correlation between higher HbA1c levels within the normal range during gestational periods and adverse birth outcomes, including preterm birth, macrosomia, and large for gestational age (LGA).

Our purpose in the present study was to evaluate the association between pregnant status and HbA1c levels among nondiabetes women using cross-sectional data from the National Health and Nutrition Examination Survey (NHANES) 2005–2016 cycles.

## 2. Methods

### 2.1. Data Sources

To provide detailed data and address important public health issues on the health conditions of the noninstitutionalized civilian population in the US, the National Health and Nutrition Examination Survey (NHANES) was set and conducted by the National Center for Health Statistics (NCHS). It was a large, nationally representative, and ongoing cross-sectional survey. We extracted data on 60936 participants from six two-year cycles of the NHANES 2005–2016 database for our study. Participants in each NHANES cycle were identified through stratified, multistage probability sampling of the noninstitutionalized population.

**Figure 1:**
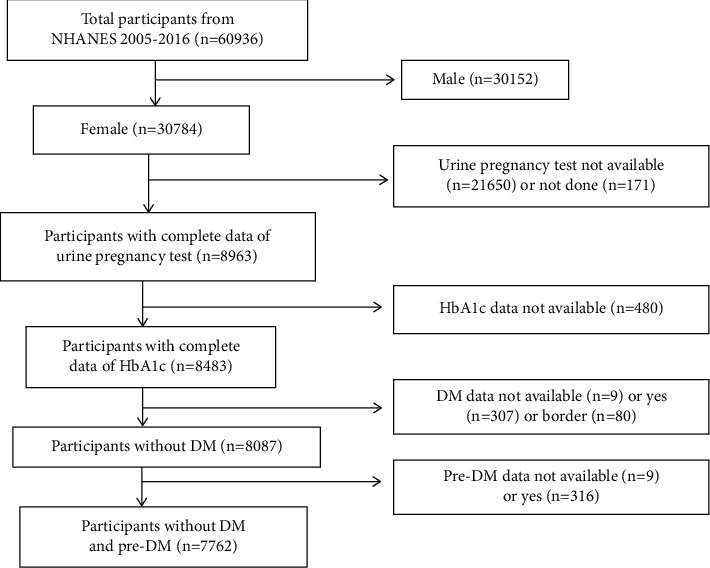
Flow chart of sample selection from the NHANES 2005–2016.

Among the 60936 participants, we excluded 30152 male participants, 21650 participants with missing urine pregnancy test data, 171 participants with no urine pregnancy test, 480 participants with missing HbA1c data, 9 participants with missing DM data, 307 participants with already DM, 80 participants with border DM, 316 participants with prediabetes, and 9 participants with missing predata. Finally, 7762 female participants without DM or prediabetes were enrolled in our analysis (Figure 1).

The NHANES survey protocol was approved by the Institutional Review Board of the National Center for Health Statistics, and written informed consent was obtained from all participants.

### 2.2. Exposure and Outcomes

Exposure was a pregnant status in this study. Pregnant status was defined according to the following criteria: positive urine pregnancy test, or being told by a doctor that they were pregnant during the interview.

The outcomes of the present study included HbA1c and serum glucose levels. As a useful clinical monitoring index, the measurement of HbA1c has been used to reflect the mean blood glucose levels in the past 8–12 weeks. HbA1cwas measured using a Tosoh Automated Analyzer HLC-723G8 (Tosoh Medics, Inc., San Francisco, CA, USA) or a Tosoh G7 automated HPLC analyzer [10]. Serum glucose (nonfasting) was measured using a Roche/Hitachi cobas C Chemistry Analyzer (Roche Diagnostics, Indianapolis, IN, USA) or a Roche/Hitachi Modular P Chemistry Analyzer.

### 2.3. Covariates

Information on age, race, smoking at least 100 cigarettes in life, and drinking at least 12 alcoholic drinks in their lifetime was obtained through self-report. Body mass index (BMI) was calculated as weight in kilograms divided by height in meters squared during the study visit. The detailed process of blood urea nitrogen, serum creatinine, total protein, serum total cholesterol, alkaline phosphatase, serum uric acid, serum sodium, serum potassium, serum phosphorus, serum calcium, hemoglobin, and platelet count was available on the NHANES website.

### 2.4. Statistical Methods

The NHANES sample weights were taken into account, as recommended by the NCHS. Statistical analyses were performed using R version 3.4.3(http://www.R-project.org) and EmpowerStats software (http://www.empowerstat.com). Statistical significance was set at *p* < 0.05. The associations of pregnant status with HbA1c and serum glucose levels were evaluated using multivariable linear regression models. According to the Strengthening the Reporting of Observational Studies in Epidemiology (STROBE) statement guidelines, three models were created in the present study: model 1, no covariates were adjusted; model 2, age and race were adjusted. In model 3, the covariates presented in Table 1 were adjusted. Subgroup analyses stratified by age and race were also performed, but the models were not adjusted for the stratification variable itself.

## 3. Results

### 3.1. Demographic Characteristics of Pregnant and Nonpregnant Nondiabetic Women

In participants without a history of diabetes, the number of pregnant women differed from that of nonpregnant women (Table 1). The average gestational period of the pregnant group in our study was 5.58 ± 2.29 months. Compared with nonpregnant women, the pregnant participants were younger, more Mexican American, and fewer other Hispanics. More pregnant women have at least 12 alcoholic drinks in their lifetime, but fewer smoked at least 100 cigarettes in their lifetime. Pregnant women had a higher BMI. Serum glucose, hemoglobin, serum uric acid, blood urea nitrogen, serum creatinine, total protein, serum sodium, serum potassium, platelet count, alkaline phosphatase, total cholesterol, and serum phosphorus were all significantly different between the two groups (*p* < 0.01 each). It is noteworthy that the average HbA1c in pregnant women in this study was 4.98 ± 0.36, lower than their counterpart in nonpregnant group with 5.26 ± 0.44 (*p* < 0.01). We also find the hemoglobin of pregnant women was 12.21 ± 1.10 g/dl, lower than that of nonpregnant women as 13.21 ± 1.20 g/dl in our study (*p* < 0.01).

### 3.2. Associations of Pregnant Status with HbA1c

In our research, urine pregnancy tests and self-reported pregnancy status were individually adopted to illuminate pregnant status. We found a negative association between pregnancy status and HbA1c.

There was a negative association between urine pregnancy test and HbA1c in all three models (model 1: *β* = −0.23, 95% CI: (−0.18 to −0.27); model 2:*β* = −0.20, 95% CI: (−0.15 to −0.24); model 3:*β* = −0.24, 95% CI: (−0.20 to −0.29)). In the subgroup analysis stratified by age, this negative association existed in all age subgroups after adjusting for confounders (age <20:*β* = −0.20, 95% CI: (−0.04 to −0.27); age ≥20, <35:*β* = −0.24 to 95% CI: (−0.20 to −0.29); age ≥35:*β* = −0.28, 95% CI: (−0.17 to −0.39)). The results are shown in Table 2. In the subgroup analysis stratified by race, negative associations steadily existed in different subgroups after adjusting for confounders (Mexican American:*β* = −0.20, 95% CI: (−0.11 to −0.29); Other Hispanic:*β* = −0.31, 95% CI: (−0.16 to −0.46); Non-Hispanic White:*β* = −0.24, 95% CI: (−0.17 to −0.31); Non-Hispanic Black:*β* = −0.21, 95% CI: (−0.12 to −0.31); Other races:*β* = −0.22, 95% CI: (−0.08 to −0.35)). The detailed information is presented in Table 3.

On the other hand, we also found a negative association between self-reported pregnancy or nonpregnancy and HbA1c in all three models (model 1:*β* = −0.22, 95% CI: (−0.18 to −0.27); model 2:*β* = −0.19, 95% CI: (−0.15 to −0.2); model 3:*β* = −0.23, 95% CI: (−0.19 to −0.28)). In the subgroup analysis stratified by age, this negative association also existed in all age groups (age <20:*β* = −0.19, 95% CI: (−0.03 to −0.26); age ≥20, <35: *β* = −0.27, 95% CI: (−0.21 to −0.32); age ≥35:*β* = −0.29, 95% CI:(−0.17 to −0.41)). The results are presented in Table 4.

### 3.3. Associations of Pregnant Status with Serum Glucose

After controlling for potential confounding factors, we found no significant associations between urine pregnancy test and serum glucose in both pregnant and nonpregnant participants (pregnant women with urine pregnancy positive: *β* = 0.00, 95% CI: (−0.09 to −0.08); pregnant women with age <20: *β* = −0.01, 95% CI: (−0.19 to 0.11); pregnant women with age ≥20, <35: *β* = −0.02, 95% CI: (−0.09 to 0.12); pregnant women with age ≥35: *β* = −0.04, 95% CI: (−0.19 to 0.27)]. The results are listed in Table 5.

## 4. Discussion

The opinion that HbA1c might be slightly lower in normal pregnancies than in normal nonpregnant women has been raised previously [11]. However, whether the pregnancy is related to HbA1c levels in nondiabetic women remains unclear. Here, our study focused on the relationship between pregnancy status and HbA1cand serum glucose levels in nondiabetic American women based on data from the NHANES database. In conclusion, our results on nondiabetic women primarily demonstrated that pregnant participants had significantly lower HbA1c levels compared with nonpregnant women, and pregnancy status was not associated with their serum glucose levels in American female participants. More importantly, HbA1c levels in the pregnant women group decreased by 0.24% compared to those in the nonpregnant group. We also find that the hemoglobin of pregnant women was 12.21 ± 1.10 g/dl, lower than that of nonpregnant women as 13.21 ± 1.20 g/dl in the study.

Moreover, we found that negative associations between pregnancy status and HbA1c existed after stratifying by age or race. Recently, a landmark study by Quenby et al. reported risk factors for miscarriage, including very young or older females (younger than 20 years and older than 35 years) [12]. Moreover, the risk of trisomy 16, as the most common cause of miscarriage, increases linearly from 20 to 40 years of age, whereas the risks of other trisomies generally show a sharp upward inflection around the age of 35 years [13]. Moreover, samples for subgroup analysis with criteria of age <20, aged ≥20 to <35, and aged >35 years in pregnant women have been widely adopted in previous studies [14, 15]. Accordingly, we reanalyzed the associations of pregnancy status with HbA1c and serum glucose levels stratified by age. In all three age subgroups, the negative association steadily existed after adjusting for confounders. However, a weaker negative association was found in women aged <20 years, and a stronger negative association was found in women aged ≥35 years after stratification by age. According to previous studies [16, 17], there are differences in HbA1c levels among different ethnic groups. Therefore, we investigated the associations between pregnancy status using a urine pregnancy test and HbA1c levels among different ethnic groups. In the analysis stratified by race, the negative associations steadily existed in different subgroups after adjusting for confounders. Further, a stronger negative association was found in other Hispanic subgroups with a 0.31% decrease, a slightly weaker negative association was found in Mexican Americans with a 0.20% decrease and non-Hispanic Black subgroup with 0.21% decrease, and a significantly weaker negative association with 0.17% decrease was found in the pregnant women group compared with the nonpregnant group.

In clinical practice, the OGTT as a reference standard is usually used to detect GDM in pregnant women. However, the OGTT test is less convenient to obtain than HbA1c, especially when screening pregnant women for GDM on a large scale. Although HbA1c is widely used as a glycemic control indicator and to diagnose DM in the general population, it is far from the HbA1c used to diagnose GDM. According to the WHO criteria, HbA1c is not currently recommended for the diagnosis of GDM. Currently, growing evidence indicates that HbA1c has the potential to detect GDM among pregnant individuals, but this remains controversial. Lai et al. [18] reported that HbA1c is weakly correlated with OGTT during late pregnancy, and it offers only limited value in diagnosing GDM among pregnant individuals when the optimal cut-off point of HbA1c was determined to be 5.0% (31 mmol/mol) for GDM diagnosis based on data of 19,261 pregnant individuals in a large Chinese tertiary hospital. Similarly, Claire and Sharon [3] conducted a systematic analysis and found that there was insufficient evidence to adopt HbA1c screening instead of OGTT for undiagnosed diabetes in the first trimester in clinical practice, which might be due to hormonal and metabolic changes occurring during pregnancy. Conversely, Kwon et al. [7] reported that HbA1c showed high sensitivity with relatively low specificity for diagnosing GDM in pregnant women and was a potential predictor of PDM. Therefore, they suggested that HbA1c might be used as a simple and less invasive alternative screening test for OGTT in patients with GDM. In contrast, Bozkurt et al. [19] took a neutral stand in this issue and declared HbA1c reflects early pathophysiological derangements in beta-cell function and glucose disposal that are characteristic of GDM development and may be useful in early risk stratification.

Researchers have reported that several factors are associated with the HbA1c levels during pregnancy. Poor sleep quality during gestational week 24 was reported to exacerbate glucose intolerance and be associated with higher HbA1c levels in pregnant women in a pilot observational study [20]. HbA1c is well known to show falsely high levels in patients with an iron-deficient state. Hashimoto and Koga [21] found that higher HbA1c levels in pregnant women without DM and prediabetes were largely affected by iron deficiency compared with nonpregnant women, but glycated albumin (GA) levels were not affected by iron content. However, iron supplementation during pregnancy does not affect HbA1c levels and has no clinical impact on moderate or severe anemia [22]. In the present study, we found that pregnancy status seems to be related to lower HbA1c levels in nondiabetic women, which might be because increased demands for iron content during pregnancy lead to relatively iron deficiency.

NHANES was designed to provide nationally representative estimates, and data collection was carried out in control of standardized protocols of the American Centers for Disease Control and Prevention so that our novel and important findings were followed by a high degree of reliability and generalizability. Moreover, we enrolled 7762 female participants without DM or prediabetes in our analysis, the largest cohort study focusing on the correlation between pregnancy status and HbA1c level from our perspective. However, there were several limitations to our current study. First, the pregnancy periods were divided into three terms of 12 weeks each: the early stage is in the first trimester, the middle stage is during gestational week 13–20, and the late stage is indicated from gestational week 20–35 [23]. The pregnant status of participants in our current study was not further divided into three stages, and HbA1c levels in different gestational stages might be very different. Second, OGTT, as the golden standard for diagnosing DM, was not included because large numbers of data from the OGTT were not described and recorded in the 2005–2016 cycles of the NHANES database, which might have caused bias when analyzing the relationship between pregnancy status and OGTT 2 hour results. Third, pregnancy status in our analysis was based on either a positive/negative urine pregnancy test or self-reported pregnant status, but not serum human chorionic gonadotropin (HCG) level, which is a much more accurate index for pregnancy. There might be a false urine pregnancy test or missing self-reported pregnant status in our data, which might cause bias. Fourth, iron deficiency has been shown to be associated with higher HbA1c levels in pregnant women. Since data on serum iron levels were not recorded, the potential confounding factor was not controlled when we analyzed the relationship between pregnancy status and HbA1c levels in nondiabetic women.

## 5. Conclusion

This study indicated that pregnant American women without diabetes and prediabetes had lower HbA1c levels than nonpregnant women. To better understand the underlying mechanism involved in explaining the relationship between pregnancy and lower HbA1c levels, more longitudinal studies are needed.

## Figures and Tables

**Table 1:** Weighted characteristics of the study sample with and without pregnancy in nondiabetes women.

	Urine pregnancy positive (*n* = 592)	Urine pregnancy negative (*n* = 7170)	*p* value
Average gestational period (months)	5.58 ± 2.29	Not available	

*Age (years)*	27.09 ± 5.80	31.03 ± 9.96	<0.001
Age groups			<0.001
<20	44 (7.43%)	921 (12.85%)	
≥20, <35	478 (80.74%)	3466 (48.34%)	
≥35	70 (11.82%)	2783 (38.81%)	
Body mass index (kg/m^2^)	28.84 ± 6.61	27.99 ± 7.48	0.007

*Race (%)*			<0.001
Mexican American	165 (27.87%)	1458 (20.33%)	
Other Hispanic	44 (7.43%)	640 (8.93%)	
Non-Hispanic White	217 (36.66%)	2684 (37.43%)	
Non-Hispanic Black	110 (18.58%)	1631 (22.75%)	
Other races including multiracial	56 (9.46%)	757 (10.56%)	

*At least 12 alcoholic drinks in a lifetime (%)*			<0.001
Yes	100 (16.89%)	845 (11.79%)	
No	128 (21.62%)	917 (12.79%)	
Not available	364 (61.49%)	5408 (75.43%)	

*Smoked at least 100 cigarettes in life (%)*			<0.001
Yes	156 (26.35%)	2015 (28.10%)	
No	392 (66.22%)	4231 (59.01%)	
Not available	44 (7.43%)	924 (12.89%)	<0.001

HbA1c (%)	4.98 ± 0.36	5.26 ± 0.44	<0.001
Serum glucose (mmol/L)	4.67 ± 0.79	4.93 ± 0.86	<0.001
Hemoglobin (g/dL)	12.21 ± 1.10	13.21 ± 1.20	<0.001
Serum uric acid (*µ*mol/L)	224.21 ± 53.54	266.86 ± 61.02	<0.001
Alkaline phosphatase (U/L)	78.52 ± 53.02	67.25 ± 31.71	<0.001
Blood urea nitrogen (mmol/L)	2.34 ± 1.00	3.69 ± 1.28	<0.001
Total cholesterol (mmol/L)	5.74 ± 1.39	4.74 ± 0.94	<0.001
Serum creatinine (*µ*mol/L)	50.44 ± 12.88	64.33 ± 19.50	<0.001
Serum phosphorus (mmol/L)	1.29 ± 0.18	1.24 ± 0.19	<0.001
Total protein (g/L)	64.44 ± 4.91	71.64 ± 4.29	<0.001
Serum sodium (mmol/L)	136.73 ± 1.87	138.77 ± 1.87	<0.001
Serum potassium (mmol/L)	3.75 ± 0.25	3.88 ± 0.28	<0.001
Platelet count (1000 cells/*µ*L)	253.80 ± 67.48	274.70 ± 68.83	<0.001

Notes: mean ± SD for continuous variables: *P* value was calculated by the weighted linear regression model. % for categorical variables: *P* value was calculated by the weighted chi-square test.

**Table 2:** Associations between urine pregnancy test and HbA1c (%) in nondiabetes women.

	Model 1 *β* (95% CI, *p*)	Model 2 *β* (95% CI, *p*)	Model 3 *β* (95% CI, *p*)
Total	Reference	Reference	Reference
Urine pregnancy negative	-−0.23 (−0.18, −0.27)	−0.20 (−0.15, −0.24)	−0.24 (−0.20, −0.29)
Urine pregnancy positive	<0.0001	<0.0001	<0.0001
Age <20	Reference	Reference	Reference
Urine pregnancy negative	−0.15 (−0.03, −0.26)	−0.15 (−0.04, −0.27)	−0.20 (−0.04, −0.27)
Urine pregnancy positive	0.0126	0.0078	<0.0001
Age ≥20, <35	Reference	Reference	Reference
Urine pregnancy negative	−0.18 (−0.14, −0.22)	−0.19 (−0.15, −0.24)	−0.24 (−0.20, −0.29)
Urine pregnancy positive	<0.0001	<0.0001	<0.0001
Age ≥35	Reference	Reference	Reference
Urine pregnancy negative	−0.25 (−0.13, −0.37)	−0.26 (−0.15, −0.38)	−0.28 (−0.17, −0.39)
Urine pregnancy positive	<0.0001	<0.0001	<0.0001

Notes: Model 1: no covariates were adjusted. Model 2: age and race were adjusted. Model 3: age, race, body mass index, smoked at least 100 cigarettes in life, drink at least 12 alcoholic drinks in a lifetime, blood urea nitrogen, serum creatinine, total protein, serum total cholesterol, alkaline phosphatase, serum uric acid, serum sodium, serum potassium, serum phosphorus, serum calcium, hemoglobin, platelet count, and serum glucose were adjusted. In the subgroup analysis stratified by age, the models are not adjusted for the stratification variable itself.

**Table 3:** Associations between urine pregnancy test and HbA1c (%) in the subgroup analysis stratified by race in nondiabetes women.

	Model 1 *β* (95% CI, *p*)	Model 2 *β* (95% CI, *p*)	Model 3 *β* (95% CI, *p*)
Mexican American	Reference	Reference	Reference
Urine pregnancy negative	−0.25 (−0.16, −0.33)	−0.20 (−0.11, −0.28)	−0.20 (−0.11, −0.29)
Urine pregnancy positive	<0.0001	<0.0001	<0.0001
Other Hispanic	Reference	Reference	Reference
Urine pregnancy negative	−0.30 (−0.15, −0.46)	−0.27 (−0.11, −0.42)	−0.31 (−0.16, −0.46)
Urine pregnancy positive	0.0001	0.0007	<0.0001
Non-Hispanic White	Reference	Reference	Reference
Urine pregnancy negative	−0.25 (−0.18, −0.31)	−0.20 (−0.14, −0.26)	−0.24 (−0.17, −0.31)
Urine pregnancy positive	<0.0001	<0.0001	<0.0001
Non-Hispanic Black	Reference	Reference	Reference
Urine pregnancy negative	−0.25 (−0.14, −0.35)	−0.19 (−0.08, −0.29)	−0.21 (−0.12, −0.31)
Urine pregnancy positive	<0.0001	0.0004	<0.0001
Other races	Reference	Reference	Reference
Urine pregnancy negative	−0.16 (−0.02, −0.30)	−0.13 (−0.00, −0.27)	−0.22 (−0.08, −0.35)
Urine pregnancy positive	0.0244	0.0560	0.0017

Notes: Model 1: no covariates were adjusted. Model 2: age was adjusted. Model 3: age, body mass index, smoked at least 100 cigarettes in life, drink at least 12 alcoholic drinks in a lifetime, blood urea nitrogen, serum creatinine, total protein, serum total cholesterol, alkaline phosphatase, serum uric acid, serum sodium, serum potassium, serum phosphorus, serum calcium, hemoglobin, platelet count, and serum glucose were adjusted.

**Table 4:** Associations between self-reported pregnancy status and HbA1c (%) in nondiabetes women.

	Model 1 *β* (95% CI, *p*)	Model 2 *β* (95% CI, *p*)	Model 3 *β* (95% CI, *p*)
Total	Reference	Reference	Reference
Nonpregnant	−0.22 (−0.18, −0.27)	−0.19 (−0.15, −0.23)	−0.23 (−0.19, −0.28)
Pregnant	<0.0001	<0.0001	<0.0001
Age <20	Reference	Reference	Reference
Nonpregnant	−0.15 (−0.03, −0.26)	−0.15 (−0.03, −0.26)	−0.19 (−0.03, −0.26)
Pregnant	0.0127	0.0124	0.0120
Age ≥20, <35	Reference	Reference	Reference
Nonpregnant	−0.18 (−0.13, −0.22)	−0.19 (−0.15, −0.23)	−0.27 (−0.21, −0.32)
Pregnant	<0.0001	<0.0001	<0.0001
Age ≥35	Reference	Reference	Reference
Nonpregnant	−0.24 (−0.13, −0.36)	−0.21 (−0.09, −0.32)	−0.29 (−0.17, −0.41)
Pregnant	<0.0001	0.0005	<0.0001

Notes: Model 1: no covariates were adjusted. Model 2: age and race were adjusted. Model 3: age, race, body mass index, smoked at least 100 cigarettes in life, drink at least 12 alcoholic drinks in a lifetime, blood urea nitrogen, serum creatinine, total protein, serum total cholesterol, alkaline phosphatase, serum uric acid, serum sodium, serum potassium, serum phosphorus, serum calcium, hemoglobin, platelet count, and serum glucose were adjusted. In the subgroup analysis stratified by age, the models are not adjusted for the stratification variable itself.

**Table 5:** Associations between urine pregnancy test and serum glucose (mmol/L) in nondiabetes women.

	Model 1 *β* (95% CI, *p*)	Model 2 *β* (95% CI, *p*)	Model 3 *β* (95% CI, *p*)
Total	Reference	Reference	Reference
Urine pregnancy negative	−0.21 (−0.13, −0.28)	−0.20 (−0.13, −0.27)	0.00 (−0.09, 0.08)
Urine pregnancy positive	<0.0001	<0.0001	0.9118
Age <20	Reference	Reference	Reference
Urine pregnancy negative	0.00 (−0.20, 0.21)	−0.02 (−0.18, 0.22)	−0.01 (−0.19, 0.11)
Urine pregnancy positive	0.9707	0.8528	1.2845
Age ≥20, <35	Reference	Reference	Reference
Urine pregnancy negative	−0.16 (−0.07, −0.25)	−0.17 (−0.08, −0.26)	−0.02 (−0.09, 0.12)
Urine pregnancy positive	0.0004	0.0002	0.7401
Age≥ 35	Reference	Reference	Reference
Urine pregnancy negative	−0.26 (−0.01, −0.50)	−0.28 (−0.03, −0.52)	−0.04 (−0.19, 0.27)
Urine pregnancy positive	0.0387	0.0275	0.7562

Notes: Model 1: no covariates were adjusted. Model 2: age and race were adjusted. Model 3: age, race, body mass index, smoked at least 100 cigarettes in life, drink at least 12 alcoholic drinks in a lifetime, blood urea nitrogen, serum creatinine, total protein, serum total cholesterol, alkaline phosphatase, serum uric acid, serum sodium, serum potassium, serum phosphorus, serum calcium, hemoglobin, platelet count, and glycohemoglobin were adjusted. In the subgroup analysis stratified by age, the models are not adjusted for the stratification variable itself.

## Data Availability

The data are publicly available on the Internet and researchers throughout the world http://www.cdc.gov/nchs/nhanes/. The original contributions presented in the study are included in the article. Further inquiries can be directed to the corresponding author.
